# Outpatient Low-Dose Initiation of Buprenorphine for People Using Fentanyl

**DOI:** 10.1001/jamanetworkopen.2024.56253

**Published:** 2025-01-24

**Authors:** Leslie W. Suen, Amy Y. Chiang, Benjamin L. H. Jones, Christine S. Soran, Michelle Geier, Hannah R. Snyder, John Neuhaus, Janet J. Myers, Kelly R. Knight, Alexander R. Bazazi, Phillip O. Coffin

**Affiliations:** 1 Division of General Internal Medicine at San Francisco General Hospital, Department of Medicine, University of California, San Francisco, San Francisco; 2 Department of Medicine, University of California, Los Angeles, Los Angeles; 3 San Francisco Department of Public Health, San Francisco, California; 4 Department of Family Medicine, University of California, San Francisco, San Francisco; 5 Department of Biostatistics and Epidemiology, University of California, San Francisco, San Francisco; 6 Department of Humanities and Social Sciences, University of California, San Francisco, San Francisco; 7 Department of Psychiatry and Behavioral Sciences, University of California, San Francisco, San Francisco

## Abstract

**Importance:**

The rise of high-potency opioids such as fentanyl makes buprenorphine initiation challenging due to the risks of precipitated withdrawal, prompting the exploration of strategies, such as low-dose initiation (LDI) of buprenorphine. However, no comparative studies on LDI outcomes exist.

**Objective:**

To evaluate outpatient outcomes associated with 2 LDI protocols of buprenorphine among individuals with opioid use disorder (OUD) using fentanyl.

**Design, Setting, and Participants:**

This cohort study analyzed data on adults with OUD who self-reported daily fentanyl use and underwent buprenorphine initiation using LDI. Data were extracted from the electronic health records of 2 substance use disorder treatment clinics using a specialty behavioral health pharmacy in San Francisco, California, from May 2021 to November 2022.

**Exposures:**

Type of LDI protocol selected by individuals: 4-day or 7-day protocol.

**Main Outcomes and Measures:**

The primary outcome was successful buprenorphine initiation, defined as self-reported LDI completion and pickup of a refill maintenance prescription, and buprenorphine retention. Logistic regression with generalized estimating equations assessed associations between LDI protocol (4-day vs 7-day) and successful initiation, adjusting for multiple attempts, age, gender identity, race and ethnicity, and housing status. Kaplan-Meier survival curves were used to estimate buprenorphine retention, and survival curves were adjusted using a fitted Cox proportional hazards regression model.

**Results:**

A total of 126 individuals (median [IQR] age, 35 [29-44] years; 90 identified as men [71%]; 26 [21%] identified as Black or African American, 20 [16%] as Latine, and 66 [52%] as White individuals) with 175 initiation attempts were included. Across attempts, 72 (41%) had a 4-day LDI protocol and 103 (59%) had a 7-day protocol. Initiation was successful in 60 attempts (34%), including 27 (38%) among 4-day protocol and 29 (28%) among 7-day protocol attempts. Buprenorphine retention rate at 28 days was 21% for a 4-day protocol and 18% for a 7-day protocol. Logistic regression found no significant differences between LDI protocols and successful initiation, while repeated LDI attempts had lower odds of success (second attempt: adjusted odds ratio [AOR], 0.30 [95% CI, 0.14-0.66]; third or more attempt: AOR, 0.22 [95% CI, 0.09-0.53]). Unadjusted and adjusted survival models did not detect a significant difference in retention between LDI protocol types.

**Conclusions and Relevance:**

This cohort study found that among people with OUD using fentanyl and attempting outpatient LDI of buprenorphine, successful buprenorphine initiation and retention rates were low. Future studies should examine interventions to improve LDI success and increase buprenorphine uptake and retention.

## Key Points

**Question:**

What outcomes are associated with 2 low-dose buprenorphine initiation protocols in the outpatient setting among people with opioid use disorder who use fentanyl?

**Findings:**

In this cohort study that included 126 adults across 175 low-dose initiation (LDI) attempts, only 60 attempts (34%) were successful in LDI of buprenorphine. No significant differences were found between protocol type (4-day vs 7-day), and buprenorphine retention rates were low.

**Meaning:**

Findings of this study suggest that in the fentanyl era, LDI has low rates of success in the outpatient setting; future studies should examine interventions to improve LDI success and increase buprenorphine uptake and retention.

## Teaser

This cohort study assesses rates of successful buprenorphine initiation and retention among adults who use fentanyl receiving a low-dose buprenorphine initiation protocol in the outpatient setting to treat opioid use disorder.

## Introduction

Increasing the uptake of buprenorphine treatment for opioid use disorder (OUD) is critical, with overdose deaths reaching alarming levels.^1,2,3^ Buprenorphine and methadone are gold standard treatments for OUD, reducing mortality by over 50%.^4,5,6^ Buprenorphine is more accessible than methadone in outpatient settings, and any physician with a US Drug Enforcement Administration license is now able to prescribe it.^7^ However, despite its proven effectiveness, buprenorphine uptake remains low, with estimates suggesting that less than 20% of individuals with OUD receive any medication treatment.^1,8^ In San Francisco, California, individuals with OUD have predominantly used fentanyl since the late 2010s.^9,10^ Despite efforts to expand treatment locally, uptake of both OUD medication options has been decreasing, with approximately 2900 unique individuals enrolled in buprenorphine in 2021 to 2538 individuals in 2022 and approximately 2286 unique individuals enrolled in methadone in 2021 to 2272 individuals in 2022.^11^

One factor in low buprenorphine uptake is people’s fear of experiencing precipitated withdrawal during initiation, particularly among those using fentanyl.^12,13^ Prior to the introduction of fentanyl, a highly potent opioid associated with a substantial proportion of overdoses,^3^ rates of successful buprenorphine initiation were relatively high,^5,6,14,15^ and although exact estimates of successful initiation in the fentanyl era are not known, experts have highlighted increased challenges.^12,16^ Buprenorphine, a partial opioid agonist with high binding affinity to the μ-opioid receptor, can displace other opioids, leading to severe precipitated withdrawal symptoms if other opioids are present in the body.^13,17^ Traditional buprenorphine initiation requires individuals to stop opioid use and experience moderate withdrawal symptoms before initiating buprenorphine to avoid precipitating withdrawal. However, fentanyl is hypothesized to accumulate in fat stores much longer than other opioids, and individuals initiating buprenorphine can still undergo severe precipitated withdrawal despite waiting until experiencing withdrawal.^18,19,20^ These challenges complicate successful initiation and highlight the pressing need to explore alternative strategies for initiating buprenorphine among individuals who use fentanyl, such as low-dose initiation (LDI) of buprenorphine.

The emerging practice of LDI of buprenorphine presents an opportunity to improve outcomes in the fentanyl era. LDI involves administering frequent doses of buprenorphine, often in low milligram doses, minimizing precipitated withdrawal risk.^17,21,22,23^ Despite growing interest in and potential advantages of LDI, the existing literature primarily is from inpatient or international settings and is limited to case series.^22,23,24,25^ Prior case series have discussed the role of LDI in outpatient settings.^25,26^ However, understanding of LDI outcomes in outpatient settings remains minimal and needed, especially as the majority of buprenorphine initiations occur in the outpatient setting. The objective of the present study was to evaluate outpatient outcomes associated with 2 LDI protocols of buprenorphine among individuals with OUD using fentanyl, in an effort to identify the potential benefits and drawbacks of this approach.

## Methods

### Design and Settings

We conducted a retrospective cohort study of individuals with OUD and self-reported daily fentanyl use who initiated buprenorphine treatment with LDI. These individuals sought care from 2 safety-net substance use disorder (SUD) treatment clinics in San Francisco—Office-Based Buprenorphine Induction Clinic (OBIC) and Bridge Clinic at San Francisco General Hospital Family Health Center—and picked up their buprenorphine prescription from the Community Behavioral Health Services (CBHS) Pharmacy. Both clinics offered SUD treatment services, including buprenorphine for OUD treatment, to uninsured or publicly insured individuals (Medicaid and Medicare) living in San Francisco.^26,27^ Both clinics sent buprenorphine prescriptions for fulfillment to CBHS Pharmacy, a behavioral health pharmacy offering specialty SUD treatment services, including bubble packaging of medications, medication counseling, and directly observed dosing. CBHS Pharmacy and OBIC are located in the same building, while the Bridge Clinic is located approximately 2 miles away. The University of California San Francisco Institutional Review Board approved this study, and written informed consent was obtained from participants. We followed the Strengthening the Reporting of Observational Studies in Epidemiology (STROBE) reporting guideline.^28^

### Participants

Eligible participants were adults aged 18 years or older with OUD and self-reported daily fentanyl use who attempted LDI of buprenorphine and picked up their buprenorphine prescription from CBHS Pharmacy between May 1, 2021, and November 30, 2022. Urine drug testing was not required to confirm opioid use given that guidelines state meeting the *Diagnostic and Statistical Manual of Mental Disorders* (Fifth Edition) (*DSM-5*) criteria for OUD is sufficient for initiating buprenorphine treatment.^29^ We excluded individuals who did not meet *DSM-5* criteria for OUD (ie, prescribed buprenorphine for chronic pain), self-reported buprenorphine or methadone use in the past 7 days, or had an intake urine drug screen (UDS) positive for buprenorphine or methadone.

### Clinic and Pharmacy Procedures

Individuals interested in initiating buprenorphine made appointments or dropped in during clinic hours. At each initiation visit, individuals provided written informed consent and met with a clinician who reviewed their substance use history and goals for treatment. Clinicians presented available options for buprenorphine initiation, including LDI protocols. People opting for LDI chose either a 4-day or a 7-day LDI protocol type. Specifics of each protocol have been described previously and are summarized in Table 1.^26,27^ Briefly, both protocols were made available in 2020, with the 7-day type using twice-a-day or three-times-a-day dose and the 4-day type offering a shorter time frame of 4-times-a-day dose. Both protocols have become popular in our clinical settings, although data have been limited to case series.^26,27^ Both protocols also used the buprenorphine monoproduct due to concerns about the buprenorphine-naloxone combination product having more adverse effects and leading to possible withdrawal. People were counseled on the risks and benefits of LDI, including the high likelihood of needing to continue using a nonprescribed full agonist opioid, such as fentanyl, and were given detailed instructions on dosing regimens, including taking buprenorphine multiple times a day.^26,27^

**Table 1.** Low-Dose Initiation Protocols for Buprenorphine Monoproduct

Day of protocol	Buprenorphine dosing^a^	
	4-d LDI protocol	7-d LDI protocol
Day 1	0.5 mg 4 times a day	0.5 mg once a day
Day 2	1 mg 4 times a day	0.5 mg twice a day
Day 3	2 mg 4 times a day	0.5 mg in morning, 1 mg in afternoon and evening
Day 4	8 mg 3 times a day^b^	2 mg twice a day
Day 5	8 mg 3 times a day^b^	3 mg twice a day
Day 6	8 mg 3 times a day^b^	4 mg twice a day
Day 7	8 mg 3 times a day^b^	8 mg 3 times a day^b^

Abbreviation: LDI, low-dose initiation.

^a^ Buprenorphine is bubble-packaged to facilitate adherence. Pharmacists also assist with splitting buprenorphine tablets prior to bubble packaging. Patients are counseled to continue use of full agonist opioids enough to stay out of withdrawal.

^b^ Final dose adjusted based on each patient’s needs.

Alternatively, individuals could have opted for traditional initiation, which involves abstaining from fentanyl use for at least 48 to 72 hours and experiencing at least moderate withdrawal before taking 8 mg or higher doses of buprenorphine. In all cases, clinicians offered adjunctive medications for withdrawal for all initiation strategies, naloxone, and counseling on harm-reduction strategies for safer drug use. Individuals then picked up their buprenorphine prescriptions in a bubble pack and any other medications from CBHS Pharmacy. CBHS Pharmacy also assisted with splitting buprenorphine tablets prior to bubble packaging and offered additional counseling on pickup.

### Data Collection and Measures

The data collection methods have been previously described.^30^ Briefly, we extracted available data from the electronic health record (EHR) in December 2022, and data spanned all LDI attempts between May 1, 2021, and November 30, 2022. Two of us conducted comprehensive, structured medical record reviews from December 2022 to March 2023, with one researcher (B.L.H.J.) conducting the initial medical record reviews and the other researcher (L.W.S.) reviewing to corroborate the data. We (B.L.H.J. and L.W.S.) also met weekly to review discrepancies and resolve issues that arose from data collection.

Data on people opting for traditional initiation were not analyzed in this study because these individuals’ clinical circumstances were fundamentally distinct from those pursuing LDI. For example, they were already in moderate to severe withdrawal or were entering a residential treatment program where ongoing fentanyl use and LDI would not be possible.

Medical record reviews collected sociodemographic characteristics (age, gender identity, race and ethnicity, primary language, primary insurance, and housing status), comorbidities (chronic pain, HIV, and mental health conditions), substance use history (prior overdose and prior OUD medication experiences), and intake UDS results if available. Housing status was extracted from the EHR and categorized as stable housing, transitional housing (single room occupancy, shelter, hotel, or staying with others), or unhoused (living in a vehicle, living on the street, or having no fixed address listed). Race and ethnicity data, also documented in the EHR, were self-reported and categorized as Black or African American, Latine,^31^ White, or other (including American Indian or Alaska Native, Asian, Native Hawaiian or Other Pacific Islander, and not reported). This variable was included in the study to identify any potential disparities by race and ethnicity. We also collected follow-up visit dates and prescription data.

The exposure of interest was the type of LDI protocol selected: either the 4-day or 7-day protocol. The primary outcome was successful buprenorphine initiation, defined as self-reported completion of the LDI protocol at a follow-up visit and pickup of a subsequent buprenorphine refill of 8 mg daily dose or higher within 1 month of the initiation date. We also examined the outcome of buprenorphine retention using prescription fill dates and days’ supply. Buprenorphine treatment discontinuation was defined as a prescription gap of 8 or more days with a subsequent new LDI prescription. For example, if an individual’s last prescription was a 2-week refill, followed by a gap of 8 days or more, and followed by a new LDI attempt, then that individual would be considered as having discontinuation at the end of the 2-week refill prescription.

### Statistical Analysis

We used descriptive statistics, including rank sum tests for continuous variables and χ^2^ tests for categorical variables, to compare differences between individuals who chose the 4-day vs 7-day protocol on their first LDI attempt. We set statistical significance at a 2-sided *P* < .05. We used logistic regression with generalized estimating equations with an independent working correlation matrix to assess the association of LDI protocol type with the primary outcome of successful initiation. We hypothesized that individuals on their second or more LDI attempt would have higher odds of successful initiation due to being more motivated or experienced during repeat attempts, and a 4-day protocol may have higher odds of successful initiation than the 7-day protocol due to individuals being less likely to drop off with a shorter duration for initiation. We included the number of attempts in both unadjusted and adjusted models to account for multiple attempts across the cohort. We also determined a priori to adjust for age, gender identity, race and ethnicity, and housing status as potential confounders of the association between LDI protocol option and successful initiation. We used robust SEs to account for clustering at the individual level.

For buprenorphine retention, we estimated Kaplan-Meier survival curves by protocol type and estimated survival curves with a Cox proportional hazards regression model at mean covariate values, using the same covariates. Individuals were followed up for a maximum of 120 days. Individuals were censored at 120 days or earlier if they experienced buprenorphine discontinuation. We compared time to treatment discontinuation by LDI protocol type using a log-rank test. All analyses were conducted in Stata 16 (StataCorp LLC).

## Results

### Participant Characteristics

A total of 126 individuals with 175 LDI attempts were analyzed. The cohort had a median (IQR) age of 35 (29-44) years and included 90 (71%) who identified as men, 33 (26%) as women, and 3 (2%) as nonbinary (Table 2). Of these participants, 26 (21%) identified as Black or African American, 20 (16%) as Latine, 66 (52%) as White, and 14 (11%) as other race and ethnicity. Housing status was mixed, with 47 (37%) of participants having stable housing, 44 (35%) having transitional housing, and 35 (28%) being unhoused. Most participants (85 [67%]) had concurrent methamphetamine use on UDS. Bivariate testing found that individuals with psychotic disorder were more likely to choose the 7-day than the 4-day LDI protocol (19 [26%] vs 6 [11%]; *P* = .03), and there were no other differences between groups.

**Table 2.** Characteristics of Individuals Using Fentanyl and Initiating Low-Dose Buprenorphine Treatment of Opioid Use Disorder^a^

Characteristic	Patients, No. (%)			*P* value^b^
	All (N = 126)	With 4-d LDI protocol (n = 54)	With 7-d LDI protocol (n = 72)	
Age, median (IQR)	35 (29-44)	35 (29-42)	36 (30-46)	.49
Gender identity				
Man	90 (71)	38 (70)	52 (72)	.26
Woman	33 (26)	16 (30)	17 (24)	
Nonbinary	3 (2)	0 (0)	3 (4)	
Race and ethnicity^c^				
Black or African American	26 (21)	10 (19)	16 (22)	.76
Latine	20 (16)	7 (13)	13 (18)	
White	66 (52)	31 (57)	35 (49)	
Other^d^	14 (11)	6 (11)	8 (11)	
Housing status				
Stable housing	47 (37)	18 (33)	29 (40)	.27
Transitional housing^e^	44 (35)	17 (32)	27 (38)	
Unhoused^f^	35 (28)	19 (35)	16 (22)	
Primary insurance				
Medicaid	94 (75)	45 (83)	49 (68)	.18
Medicare	8 (6)	1 (2)	7 (10)	
Uninsured	8 (6)	4 (7)	4 (6)	
Other or unknown	16 (13)	4 (7)	12 (16)	
Comorbidities				
Chronic pain	33 (26)	18 (33)	15 (21)	.11
HIV	10 (8)	3 (6)	7 (10)	.54
Anxiety disorder	56 (44)	21 (39)	35 (49)	.28
Depression	53 (42)	24 (44)	29 (40)	.64
Bipolar disorder	21 (17)	8 (15)	13 (18)	.63
Psychotic disorder	25 (20)	6 (11)	19 (26)	.03
Self-reported opioids used in addition to fentanyl				
Opioid pills	2 (2)	0 (0)	2 (3)	.22
Heroin	31 (25)	12 (22)	19 (26)	.59
Baseline UDS result				
Benzodiazepines	18 (14)	6 (11)	12 (17)	.30
Cannabis	46 (36)	21 (39)	25 (35)	.51
Cocaine	38 (30)	20 (37)	18 (25)	.25
Opiates	22 (17)	12 (22)	10 (14)	.30
Oxycodone	2 (2)	0 (0)	2 (3)	.24
Methamphetamine	85 (67)	37 (69)	48 (67)	.47
No baseline UDS result available	14 (11)	4 (7)	10 (14)	.25
Prior overdose	83 (66)	38 (70)	45 (62)	.30
Buprenorphine treatment experienced	87 (71)	34 (64)	53 (76)	.36
Primary route				
Injection	13 (10)	6 (11)	7 (10)	.86
Inhalational: vaporization	99 (79)	41 (76)	58 (81)	
Intranasal: snorting	5 (4)	2 (4)	3 (4)	
Unknown	9 (7)	5 (9)	4 (6)	

Abbreviations: LDI, low-dose initiation; UDS, urine drug screen.

^a^ Patients were categorized based on the LDI protocol chosen at their first buprenorphine initiation attempt.

^b^ Calculated using rank sum test for continuous variables (age) and χ^2^ tests for categorical variables (all others).

^c^ Self-reported and documented in the electronic health record.

^d^ Included American Indian or Alaska Native, Asian, Native Hawaiian or Other Pacific Islander, and not reported.

^e^ Included those who reported currently staying in a single room occupancy, shelter, or hotel or staying with others.

^f^ Included those who reported currently staying on the street or in a vehicle or having no fixed address listed.

### Buprenorphine Initiation Outcomes

**Figure 1.**
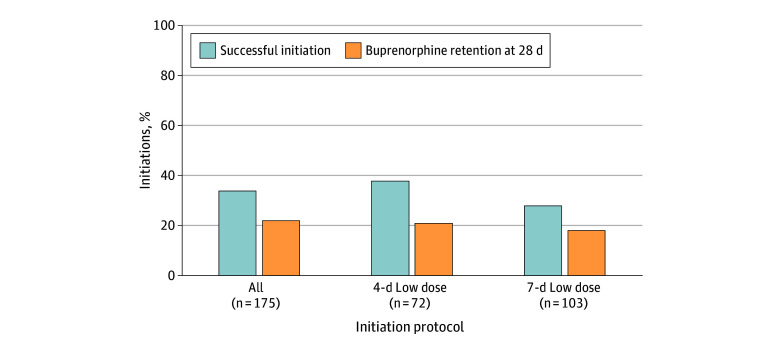
Treatment Outcomes of 4-Day vs 7-Day Low-Dose Initiation Attempts of Buprenorphine Among Individuals With Opioid Use Disorder Using Fentanyl Outcomes include successful initiation and buprenorphine retention rates at 28 days after initiation.

Across 175 LDI attempts, 72 attempts (41%) were for the 4-day protocol and 103 attempts (59%) were for the 7-day protocol (Figure 1). The reason cited for all LDI attempts was the desire to avoid any or precipitated withdrawal, and the reasons for choosing a 7-day vs a 4-day protocol were not consistently documented. At follow-up visits, 27 attempts (38%) with a 4-day protocol and 29 attempts (28%) with a 7-day protocol achieved successful buprenorphine initiation. Overall, LDI was successful in 60 attempts (34%). Buprenorphine retention rate at 28 days was 21% for the 4-day and 18% for the 7-day LDI protocol. Overall, 39 attempts (22%) were retained on buprenorphine at 28 days.

Of the 126 individuals in this study, 43 (34%) were lost to follow-up after their initial visit. Eighty-nine of 126 people (71%) had only 1 LDI attempt, of which 28 were successful on the first attempt (eTable 1 in Supplement 1). The remaining 37 participants (29%) had 2 attempts or more, of which 15 chose a different LDI protocol on a successive attempt. Fourteen of the 37 participants had successful initiation on a successive attempt.

In logistic regression models for successful initiation (Table 3), there were no statistically significant differences between the 4-day and 7-day LDI protocols in unadjusted (odds ratio [OR], 1.33; 95% CI, 0.74-2.43) and adjusted models (adjusted OR [AOR], 1.52; 95% CI, 0.82-2.81). Repeated attempts were associated with lower odds of successful initiation compared with first attempts (second attempt: OR, 0.40 [95% CI, 0.19-0.77] and AOR, 0.30 [95% CI, 0.14-0.66]; third or more attempt: OR, 0.20 [95% CI, 0.07-0.55] and AOR, 0.22 [95% CI, 0.09-0.53]). Furthermore, compared with people with stable housing, those with transitional housing (AOR, 0.32; 95% CI, 0.14-0.73) or unhoused (AOR, 0.39; 95% CI, 0.17-0.91) had lower odds of successful attempts.

**Table 3.** Association Between 7-Day vs 4-Day Low-Dose Initiation Attempts and Successful Buprenorphine Initiation (N = 175)

	OR (95% CI)	
	Unadjusted	Adjusted
Protocol		
7-d LDI	1 [Reference]	1 [Reference]
4-d LDI	1.33 (0.74-2.43)	1.52 (0.82-2.81)
LDI attempts		
First	1 [Reference]	1 [Reference]
Second	0.40 (0.19-0.77)	0.30 (0.14-0.66)
Third or more	0.20 (0.07-0.55)	0.22 (0.09-0.53)
Age	NA	0.97 (0.93-1.01)
Gender identity		
Woman	NA	1 [Reference]
Man	NA	1.49 (0.70-3.17)
Nonbinary	NA	5.71 (1.32-24.68)
Race and ethnicity		
Black or African American	NA	1 [Reference]
Latine	NA	1.18 (0.35-3.95)
White	NA	2.32 (0.88-6.08)
Other	NA	2.17 (0.70-6.73)
Housing status		
Stable housing	NA	1 [Reference]
Transitional housing	NA	0.32 (0.14-0.73)
Unhoused	NA	0.39 (0.17-0.91)

Abbreviations: LDI, low-dose initiation; NA, not applicable; OR, odds ratio.

**Figure 2.**
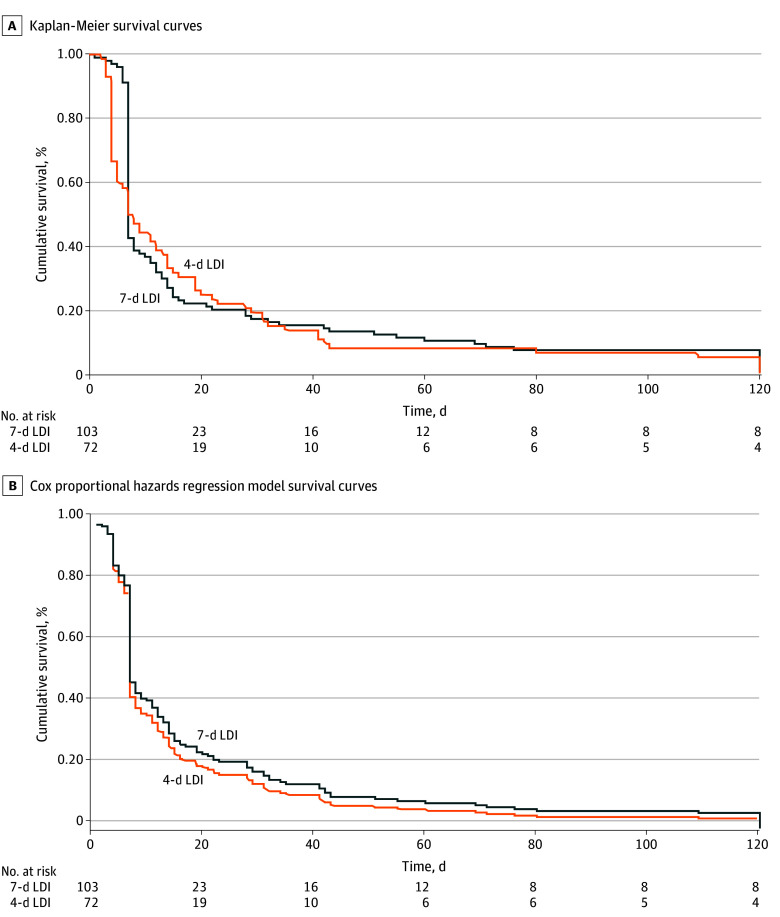
Survival Analyses of Low-Dose Initiation (LDI) Protocols and Time to Buprenorphine Treatment Discontinuation

For buprenorphine retention, Kaplan-Meier curves showed slightly higher probabilities of retention in LDI attempts with the 7-day protocol compared with the 4-day protocol, although there were no statistically significant differences between the 2 protocols (log-rank test *P* = .21) (Figure 2A). The adjusted survival curve using a fitted Cox proportional hazards regression model found similar results (Figure 2B), with no statistically significant differences in treatment discontinuation between the 2 protocols (eTable 2 in Supplement 1).

## Discussion

To our knowledge, this retrospective cohort study was the largest to evaluate outcomes of LDI of buprenorphine attempts in the outpatient setting among people with OUD using fentanyl. Successful completion of buprenorphine initiation was low at only 34%, and only 22% of attempts were retained on buprenorphine at 28 days. These rates are lower than those in prior studies in the prefentanyl era among people using heroin or prescription opioids, which reported more than 90% rates of successful initiation.^5,6,14,15^ Lower successful initiation rates for buprenorphine compared with heroin are expected given the pharmacologic challenges associated with chronic fentanyl use and the higher risk for precipitated withdrawal,^32^ although the significantly lower rate of success highlights the need to improve buprenorphine care and retention.

Furthermore, success rates of LDI in outpatient settings were substantially lower in this study than other studies examining LDI use in the hospital. Four single-site retrospective studies reported more than 68% to 80% completion among patients who underwent LDI in the hospital.^22,25,33,34^ Potential reasons for these findings are that hospitals are more controlled than outpatient clinics, with timed dosing of buprenorphine administered by hospital staff, minimizing room for error. Hospitals also provide a regulated, predictable supply of full agonist opioids to treat withdrawal symptoms, with the support of clinicians and nurses who could adjust medications as needed. The expectation that outpatients continue to use unprescribed full agonists, such as fentanyl, during LDI may be a factor in this discrepancy; with an unreliable, inconsistent drug supply, outpatients using fentanyl may not be able to control their opioid requirements reliably.^26^

Allowing individuals access to a safe supply of opioids, such as methadone or oral hydromorphone, potentially plays a role in successful completion, although prescription of full agonist opioids to treat OUD withdrawal is illegal in the US per the Controlled Substance Act.^35^ One exception is the 72-Hour Rule introduced by the Drug Enforcement Administration in 2021, which allows for the dispensing of a 72-hour supply of controlled substances (most commonly methadone) to treat withdrawal symptoms while a person awaits linkage to OUD treatment.^36,37,38^ This pathway, therefore, allows clinics to consider dispensing a short course of methadone or other long-acting full agonist opioid to support a 4-day LDI protocol. This practice has been reported from a clinic in Boston, Massachusetts, although its associated outcomes are not yet known.^39^

Individuals using LDI may need more frequent support from clinicians on how to manage withdrawal symptoms. LDI protocols take several days to complete, and prior work has highlighted higher-than-expected withdrawal symptoms during LDI, with 31% of patients experiencing some withdrawal, 21% of which was mild.^30^ Additionally, adjunctive medications were used less than half the time.^30^ Clinicians may, therefore, normalize some withdrawal during counseling and consider increasing their prescribing of adjunctive medications. Clinics can also offer support through closer follow-up calls or a contact person to call for issues. Bubble packaging of medications facilitates adherence, but LDI protocols remain complex with room for error.

Buprenorphine retention at 28 days was also particularly low for LDI attempts. Prior studies of buprenorphine among people using heroin have found 28-day retention to be closer to 37% to 60%.^5,40,41,42^ Some have hypothesized that with high rates of fentanyl use, higher doses of buprenorphine (≥24 mg) may be needed to treat cravings effectively, and some people using fentanyl may have improved retention with methadone.^43,44,45^ Additionally, unlike in traditional initiation, where people move from experiencing moderate withdrawal to immediate relief with at least 8 mg initial buprenorphine doses, those undergoing LDI do not experience a sudden improvement in symptoms after buprenorphine. This lack of timely reinforcement may also be associated with low LDI success. Future qualitative studies should investigate patient and clinician experiences with LDI, identifying the barriers and challenges to successful initiation and evaluating various interventions, such as incentives for completion, akin to contingency management to engender favorable associations with buprenorphine use.^46^ Adaptive trial designs and partially randomized preference trials may also have a role in incorporating patient and clinician treatment preferences and evaluating intervention effectiveness.^47,48^

We found insufficient evidence to conclude which type of LDI protocol was associated with increased successful initiation, especially given that the power to detect a difference between protocols may have been too low. We also found that individuals who lacked stable housing had lower odds of success. This population faces numerous challenges with LDI, including high rates of medication theft and lack of a safe, reliable place for using fentanyl or handling withdrawal symptoms.^49^ Additional support is especially needed to increase successful LDI of buprenorphine among individuals experiencing homelessness, who face high rates of overdose-related mortality.^42,49^

Contrary to our hypothesis, people had lower odds of successful initiation with repeated attempts. Despite this finding, those who return to the clinic after an unsuccessful first LDI attempt and desiring a second attempt should be allowed to do so to promote autonomy, with clinicians assisting in troubleshooting. However, those with 2 or more unsuccessful LDI attempts may be advised to try alternative strategies. For example, the mechanism of action of injectable buprenorphine formulations offers a slow onset of medication that may mimic LDI protocols, with similarly lower risk of precipitated withdrawal.^50,51^ Direct initiation using newer, weekly formulations of injectable buprenorphine (eg, CAM2038) without requiring moderate initial withdrawal may be more appropriate for people who are unable to tolerate complex LDI instructions.^51^ Evidence on this practice, however, is relatively nascent and requires further study.^51,52,53^

### Strengths and Limitations

This study has several strengths. To our knowledge, it was the largest study to report LDI outcomes in the outpatient setting and was the first study to compare LDI protocols in assessing treatment outcomes. It also described several measures of follow-up, including follow-up visits and prescription data.

This study also has several limitations. First, retrospective data collection and participants’ selection of LDI protocol can introduce bias, limiting our ability to compare outcomes across protocols. Second, while the point estimate for the AOR for successful initiation between LDI protocols was 1.52, the 95% CI (0.82-2.81) included the null, and the small sample size may have had reduced power to detect an effect. In the future, larger studies are needed to conclude the comparative effectiveness of LDI protocols. Third, the findings may not be generalizable to other settings. OBIC and the Bridge Clinic offer specialized SUD treatment, with OBIC being located in the same building as the CBHS Pharmacy, which provides bubble packaging for LDI. The patient population also has several unique features that further limit generalizability, including high levels of buprenorphine experience, inhaled fentanyl use, and concomitant methamphetamine use. Inhaled fentanyl use compared with injection may be associated with higher opioid tolerance due to more frequent use and alteration of physiologic responses to buprenorphine. Concomitant methamphetamine use may also be a factor in decreased successful buprenorphine initiation; future studies could examine cotreating stimulant use disorder to increase the buprenorphine retention rate.^54^ Fourth, it is unclear what role the clinician had in patient selection of LDI type, as clinicians may have affected patient choice, and future research should identify how clinicians guide LDI protocol selection.

## Conclusions

Despite the lack of robust clinical evidence supporting LDI effectiveness, clinical practice has outpaced scientific evidence due to the threat of escalating overdose deaths. LDI has become an increasingly common strategy for initiating buprenorphine treatment among a cohort of publicly insured or uninsured persons using fentanyl. However, successful LDI completion in outpatient settings remains low, and successive attempts at LDI yield diminishing returns. Future studies should examine interventions to improve LDI success and increase buprenorphine uptake and retention.

## Notes

Supplement 1. **eTable 1.** Number of Attempts Per Patient and Successful Buprenorphine Initiation on Most Recent Attempt for 4-Day and 7-Day Low Dose Initiation Attempts Among Individuals With Opioid Use Disorder Using Fentanyl **eTable 2.** Adjusted Survival Model Using Fitted Cox Proportional Hazards Comparing Type of Low Dose Initiation Protocols and Time to Buprenorphine Treatment Discontinuation (n = 175)

Supplement 2.
Data Sharing Statement
