# Supplementary material for: Outpatient Low-Dose Initiation of Buprenorphine for People Using Fentanyl
Source: JAMA Netw Open. 2025 Jan 24;8(1):e2456253. doi: 10.1001/jamanetworkopen.2024.56253 (PMC11762237; doi:10.1001/jamanetworkopen.2024.56253)
Supplement: Supplement 1. — eTable 1. Number of Attempts Per Patient and Successful Buprenorphine Initiation on Most Recent Attempt for 4-Day and 7-Day Low Dose Initiation Attempts Among Individuals With Opioid Use Disorder Using Fentanyl eTable 2. Adjusted Survival Model Using Fitted Cox Proportional Hazards Comparing Type of Low Dose Initiation Protocols and Time to Buprenorphine Treatment Discontinuation (n = 175) [file jamanetwopen-e2456253-s001.pdf]

Supplemental Online Content

Suen LW, Chiang AY, Jones BLH, et al. Outpatient low-dose initiation of buprenorphine for people using fentanyl. *JAMA Netw Open*. 2025;8(1):e2456253.
doi:10.1001/jamanetworkopen.2024.56253

eTable 1. Number of Attempts Per Patient and Successful Buprenorphine Initiation on Most Recent Attempt for 4-Day and 7-Day Low Dose Initiation Attempts Among Individuals With Opioid Use Disorder Using Fentanyl

eTable 2. Adjusted Survival Model Using Fitted Cox Proportional Hazards Comparing Type of Low Dose Initiation Protocols and Time to Buprenorphine Treatment Discontinuation (n=175)

This supplemental material has been provided by the authors to give readers additional information about their work.

© 2025 Suen LW et al. *JAMA Network Open.*

eTable 1. Number of Attempts Per Patient and Successful Buprenorphine Initiation on Most Recent Attempt for 4-Day and 7-Day Low Dose Initiation Attempts Among Individuals With Opioid Use Disorder Using Fentanyl

Exact Number of Attempts Per Patient	Total Patients (n=126)	4-day Initiations Patients (n=54)	7-Day Initiations Patients (n=72)
**Had 1 attempt only**	28/89 (31%)	12/39 (31%)	16/50 (32%)
**Had 2 attempts**	11/25 (44%)	4/9 (44%)	7/16 (43%)
**Had 3 attempts**	3/10 (30%)	1/4 (25%)	2/6 (33%)
**Had 4 attempts**	0/1 (0%)	0/1 (0%)	-
**Had 5 attempts**	-	-	-
**Had 6 attempts**	0/1 (0%)	0/1 (0%)	-

© 2025 Suen LW et al. *JAMA Network Open.*

eTable 2. Adjusted Survival Model Using Fitted Cox Proportional Hazards Comparing Type of Low Dose Initiation Protocols and Time to Buprenorphine Treatment Discontinuation (n=175)[a]

	Adjusted Hazard Ratio (95% CI)
**Protocol**	
7-day Low Dose Initiation	Ref
4-day Low Dose Initiation	1.14 (0.85, 1.54)
**Attempts**	
1st attempt	Ref
2nd attempt	1.19 (0.83, 1.69)
3rd attempt	1.11 (0.77, 1.61)
4th attempt	1.22 (0.68, 2.21)
6th attempt	4.33 (2.41, 7.79)
**Age**	1.01 (1.00, 1.03)
**Gender Identity**	
Women	Ref
Men	1.03 (0.76, 1.40)
Non-binary	0.69 (0.43, 1.10)
**Race/ethnicity**	
Black/African American	Ref
Latine	1.24 (0.75, 2.05)
White	0.80 (0.50, 1.28)
Other	0.82 (0.48, 1.39)
**Housing**	
Stably Housed	Ref
Transitional or temporary housing	1.73 (1.24, 2.41)
Unhoused	1.36 (0.90, 2.07)

[a] Buprenorphine treatment discontinuation defined as a gap in buprenorphine treatment prescriptions of 8 days or more with subsequent new initiation attempt, based on prescription fill dates and days' supply.

© 2025 Suen LW et al. *JAMA Network Open.*
