# Supplementary material for: Outpatient Low-Dose Initiation of Buprenorphine for People Using Fentanyl
Source: JAMA Netw Open. 2025 Jan 24;8(1):e2456253. doi: 10.1001/jamanetworkopen.2024.56253 (PMC11762237; doi:10.1001/jamanetworkopen.2024.56253)
Supplement: Supplement 2. — Data Sharing Statement [file jamanetwopen-e2456253-s002.pdf]

Data Sharing Statement

Suen. Outpatient Low-Dose Initiation of Buprenorphine for People Using Fentanyl. *JAMA Netw Open*. Published January 24, 2025. doi:10.1001/jamanetworkopen.2024.56253

Data
Data available: No

Additional Information
Explanation for why data not available: Data involves a relatively small sample size and will not be provided to protect the anonymity and protected health information of individuals.
